# LncRNA DANCR counteracts premature ovarian insufficiency by regulating the senescence process of granulosa cells through stabilizing the interaction between p53 and hNRNPC

**DOI:** 10.1186/s13048-023-01115-3

**Published:** 2023-02-18

**Authors:** Di Sun, Yining Wang, Ningxia Sun, Zhongxin Jiang, Ziyuan Li, Liang Wang, Fu Yang, Wen Li

**Affiliations:** 1 Center for Reproductive Medicine & Fertility Preservation Program, International Peace Maternity and Child Health Hospital, School of Medicine, Shanghai Jiao Tong University, Shanghai, 200030 China; 2 The Center of Reproductive Medicine, Shanghai Changzheng Hospital, Naval Medical University, Shanghai, 200003 China; 3 The Department of Medical Genetics, Naval Medical University, Shanghai, 200433 China

**Keywords:** Premature ovarian insufficiency, DANCR, Granulosa cell aging, hNRNPC, p53

## Abstract

**Background:**

Premature ovarian insufficiency (POI) is one of the common women reproductive endocrine diseases which adversely impacts female fertility, but the etiology and pathogenesis still remain elusive. Recently increasing researches focus on the roles of lncRNA in POI. LncRNA DANCR was involved in cell differentiation and multiple cancers. It’s highly expressed in ovary while the role of DANCR in POI is still unknown.

**Results:**

Here, we identify a new POI related lncRNA *DANCR*, which negatively contributes to ovarian granulosa cells aging and follicular atresia. DANCR is proved to be decreasingly expressed in POI patients’ granulosa cells. Additionally, *Dancr* knockout (*Dancr*^*−/−*^) mice were constructed and characterized with POI phenotypes and fertility decline, compared with *Dancr*^+*/*+^ mice. Further, in vitro experiments indicated that *DANCR* knockdown in granulosa cells led to cell aging and series of aging-related changes including proliferation inhibition, cell cycle G1 arrest and DNA damage. Mechanism research revealed DANCR binds with hNRNPC and p53, while *DANCR* knockdown attenuates the binding of hNRNPC and p53, thus enhancing protein level of p53 and promoting granulosa cells aging significantly.

**Conclusion:**

The newly identified lncRNA DANCR inhibits p53-dependent granulosa cells aging by regulating hNRNPC-p53 interaction, and eventually counteracting POI. This provides new insights into the pathogenesis of POI and provides a potential target for future diagnosis and treatment.

**Supplementary Information:**

The online version contains supplementary material available at 10.1186/s13048-023-01115-3.

## Background

POI is characterized with primary or secondary amenorrhea before the age of 40, accompanied with increased follicle stimulating hormone (FSH) level, decreased estrogen level and various symptoms of low estrogen. A recent study indicates the global incidence of POI has risen from about 1% to 3.7% [1, 2], which impairs women’s reproductive health and fertility seriously. POI is highly heterogeneous in etiology, and the pathogenesis is very complex and elusive [3]. Increased follicle atresia and accelerated follicle depletion is one of the key processes in POI [4, 5]. Granulosa cells (GCs) are key auxiliary cells that regulates the follicle development and dysfunction/atresia [6–8], and mounting evidence suggests abnormal proliferation/apoptosis and aging of GCs could lead to POI or ovary aging [9–11]. And the pool of oocytes is lost indirectly as a consequence of GCs apoptosis [12].

Long non-coding RNAs (lncRNAs) are classified as noncoding RNAs longer than 200 nucleotides, which play a vital role in the regulating of protein-coding genes expression via chromatin modification, transcription, mRNA decay, protein subcellular localization and other key processes [13]. Increasing evidences indicate that lncRNAs are implicated in folliculogenesis, GCs dysfunction and female reproductive disorders including POI [14]. A study revealed 20 lncRNAs were differentially expressed in cortical tissues of POI ovaries, and these differentially expressed transcripts were associated with follicular development and granulosa cell function [15]. Recently, a variety of lncRNAs have been identified to regulate the proliferation, differentiation, apoptosis and DNA damage repair of GCs through different mechanisms, thereby participating in the pathogenesis of POI. [16–21].

Differentiation antagonizing non-protein coding RNA (DANCR), a single 855-bp RNA transcript, was first reported as a negative regulator of cell differentiation and regulates global gene expression associated with epidermal differentiation [22]. Emerging investigation suggested that DANCR was more like a controller of differentiation orientation, such as promoting chondrogenesis and inhibiting osteoblastic differentiation of bone marrow mesenchymal stem cell (BMSCs) [23]. Also DANCR was involved in promoting inflammation and osteoclastogenesis in fracture and osteoarthritis [23]. In addition, DANCR has been widely proved to play a critical role in multiple cancers development involving cell proliferation, apoptosis, metastasis and other tumor biology processes [24, 25]. Linn et al. [26] performed RNA-seq regarding to 27 human different tissues, interestingly, DANCR was relatively highly expressed in ovary compared with other human tissues. However, there is almost no study about the function of DANCR in ovary tissue, especially in the key follicle development/dysfunction and related diseases like POI. Here, we speculate that DANCR may be implicated in the process of POI by affecting the physiological functions of follicle or GCs.

We investigated the role and regulation mechanism of DANCR in POI. The results demonstrated that DANCR had been significantly down-regulated in granulosa cells of POI patients compared with normal subjects. Additionally, DANCR knockout mice presented an attenuated fertility and a POI phenotype such as increased follicle atresia and endocrine disorders. Further, in vitro experiments indicated that, knockdown of DANCR negatively regulated the interaction between hNRNPC and p53. Accumulation of p53 protein resulted in granulosa cell aging, follicular atresia and accelerated POI.

## Results

### *DANCR* is associated with POI and knockdown of *DANCR* induces granulosa cells aging

**Fig. 1:**
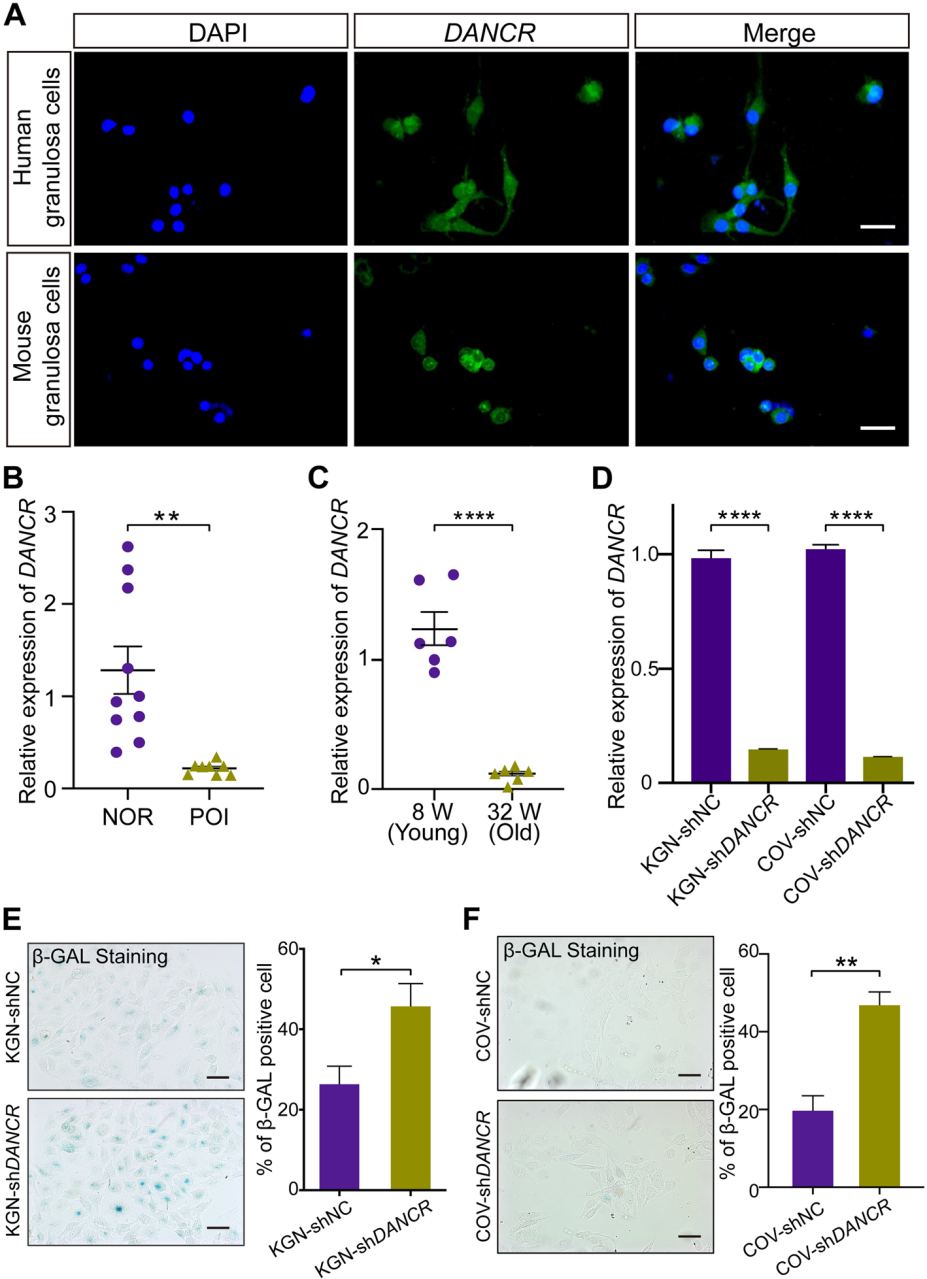
DANCR is decreasingly expressed in POI and DANCR knockdown induces ovarian granulosa cells aging. **A,** FISH staining of DANCR in human and mouse ovarian granulosa cells. **B,** The qPCR result of DANCR expression in granulosa cells of normal women (NOR group) and POI patients. **C,** Dancr expression of primary granulosa cells was qualified using qPCR in the old (32 weeks) mice and young (8 weeks) mice. **D,** DANCR knockdown efficiency in KGN and COV434 granulosa cell lines. SA-β-Gal staining in KGN cells **(E)** and COV343 cells **(F)**, the blue indicates aging cells and the percentage of aging cells is quantified. * *p* < 0.05, **p < 0.01, **** *p* < 0.0001. Scale bar: 50 μm

To identify the expression of DANCR in ovary, human and mice primary granulosa cells were collected and carried with FISH, indicating the expression of DANCR in GCs (Fig. 1A). The function of ovarian granulosa cells is bound up with follicle development and POI process, knockdown of DANCR might influence granulosa cells, leading to follicle atresia. POI develops in parallel with characteristics of granulosa cells aging [27]. Based on our hypothesis mentioned above that DANCR may be implicated in the process of POI, we detected the DANCR level in granulosa cells of POI patients and normal women. The clinical basic characteristics of the two groups are not significantly different, except for anti-Müllerian hormone (AMH), antral follicle count (AFC) and retrieved oocytes count (Table 1). Remarkably, the expression of DANCR was significantly reduced in POI patients’ ovarian granulosa cells (Fig. 1B). Additionally, Dancr expression was distinctly decreased in old (32 weeks) C57BL/6 mice’s primary granulosa cells compared with the young (8 weeks) (Fig. 1C). *DANCR* in human granulosa tumor cell lines (KGN and COV434) was silenced via lentivirus transfection and the efficiency was confirmed by qPCR (Fig. 1D). The senescence-associated β-galactosidase (SA-β-Gal) staining found that *DANCR*-knockdown resulted in a significantly higher percentage of aging granulosa cell (KGN and COV434) in vitro (Fig. 1E and F). Altogether, these results indicated that DANCR was expressed in GCs and decreased DANCR was associated with POI and ovarian granulosa cells aging.

**Table 1:** Clinical characteristics of IVF patients

	Normal	POI^a^	*P* value
Age-yr	31.67 ± 1.189	30.83 ± 0.9757	0.2967
Body mass index (BMI)	20.98 ± 0.9168	21.94 ± 1.142	0.258
Hormones			
AMH (ng/ml)	5.572 ± 0.3917	0.4083 ± 0.06336	< 0.0001
FSH (mIU/ml)	5.929 ± 0.344	8.365 ± 0.9541	0.0126
E_2_ (pg/ml)	39.75 ± 5.201	116.4 ± 73.02	0.1532
Follicles			
Antral follicle count (AFC)	11.83 ± 1.364	3.917 ± 0.8744	< 0.0001
Number of retrieved oocytes	7.25 ± 0.9056	2.167 ± 0.716	0.0001

^a^POI patients have been treated with hormone therapy

### Knockdown of DANCR induces granulosa cells aging-related changes in vitro

**Fig. 2:**
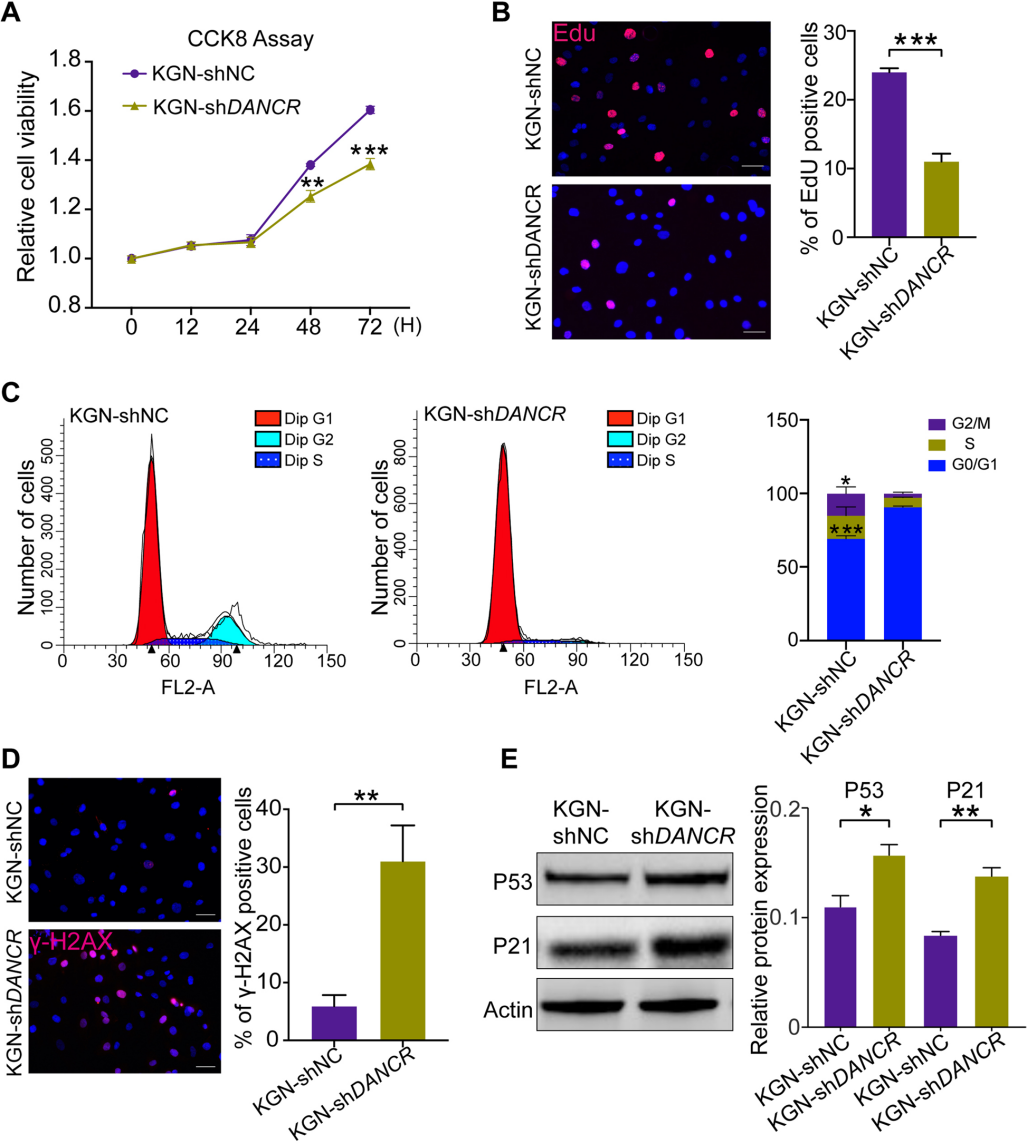
Knockdown of DANCR inhibits proliferation and promotes cell aging-related changes in KGN cells. CCK-8 **(A)** assays and EdU staining **(B)** were performed to investigate cell proliferation capability with DANCR knockdown. **C,** Flow cytometry was performed to detect the cell cycle, indicating the proportion of G1 phase cells in sh-DANCR group is significantly higher than that of the control group.** D,** γ-H2AX immunefluorescence staining indicates cells with DNA damage (red fluorescence) and quantify analysis was performed. **E,** Aging-related proteins p53 and p21 levels between the sh-DANCR and control. *p < 0.05, ** *p* < 0.01, *** *p* < 0.001. Scale bar: 50 μm

**Fig. 3:**
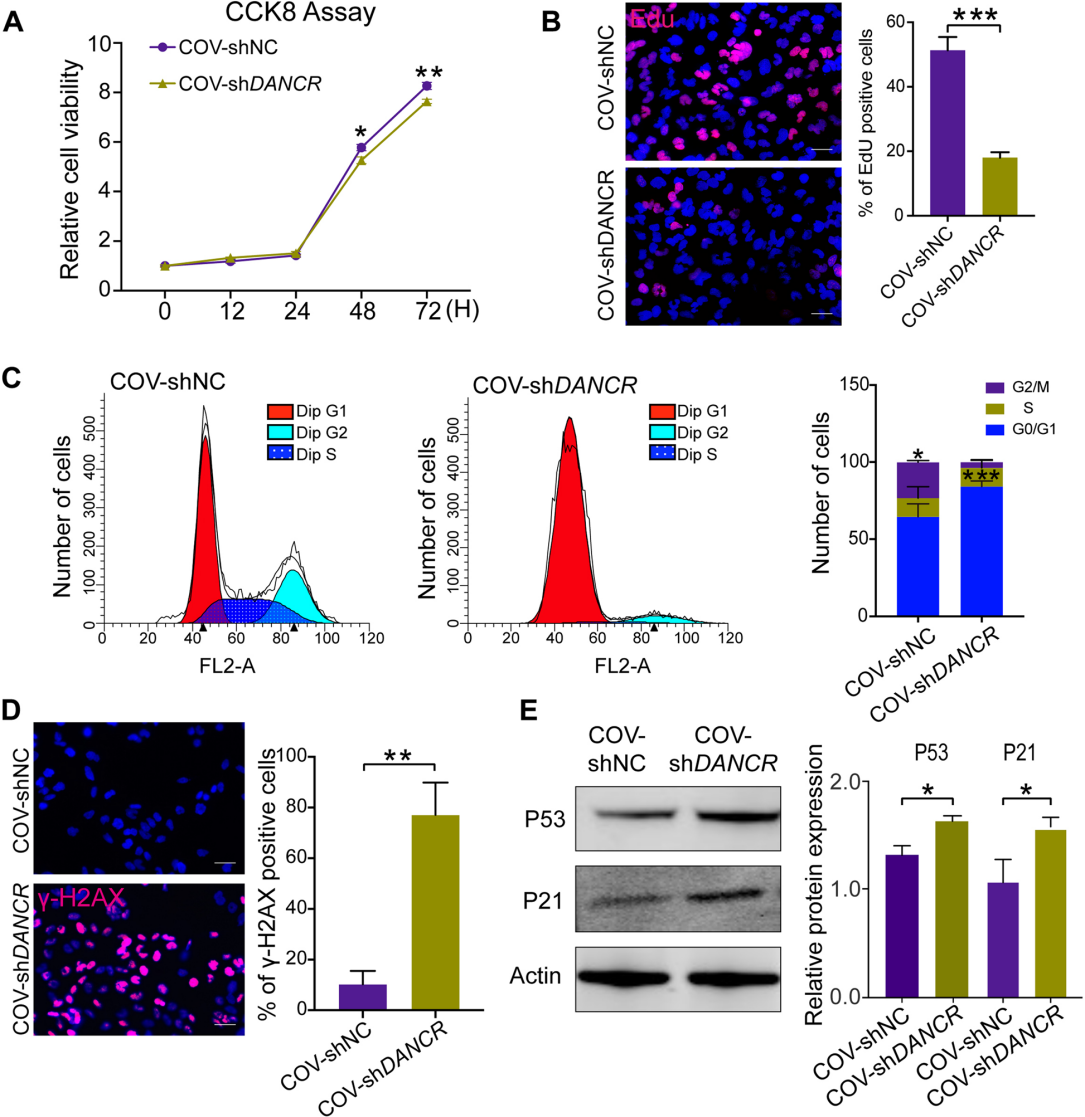
Knockdown of DANCR inhibits proliferation and promotes aging-related changes in COV434 cells. CCK-8 assays **(A)** and EdU staining **(B)** were performed to investigate cell proliferation capability with DANCR knocked down. **C,** Flow cytometry was performed to detect the cell cycle, indicating that the proportion of G1 phase cells in sh-DANCR group is significantly higher than that of the control group. **D,** γ-H2AX immunefluorescence staining indicates cells with DNA damage (red fluorescence) and quantify analysis was performed. **E,** Aging-related proteins p53 and p21 levels between the sh-DANCR and control. *p < 0.05, ** *p* < 0.01, *** *p* < 0.001. Scale bar: 50 μm

We further investigated the influence of DANCR to aging-related function changes in KGN and COV434 cell lines. The CCK-8 assays indicated cell viability of shDANCR group was visibly reduced at 48 h and 72 h compared with the control (Figs. 2A and 3A). Meanwhile, with knockdown of DANCR, the percentage of EdU-positive cells was significantly lower than the control (Figs. 2B and 3B). Both of the experiment results suggested DANCR is critical for granulosa cells proliferation. Interestingly, further experiments showed that DANCR-knockdown granulosa cells underwent G1 arrest (Figs. 2C and 3C) and more DNA damage (Figs. 2D and 3D), which both were one of the indications of cell aging. P53 functioned as a transcription factor, plays a central role in cellular senescence, governing the cell cycle arrest and senescence-associated secretory phenotype (SASP) independently. Also, p21, the p53 target gene, induces a senescent-like growth arrest [28–30]. In our study, knockdown of DANCR significantly upregulated p53 and p21 expression (Figs. 2E and 3E), which demonstrated silencing DANCR accelerate granulosa cell aging in another point of view.

### Knockout of *Dancr* impairs fertility and leads to a POI phenotype in mice

**Fig. 4:**
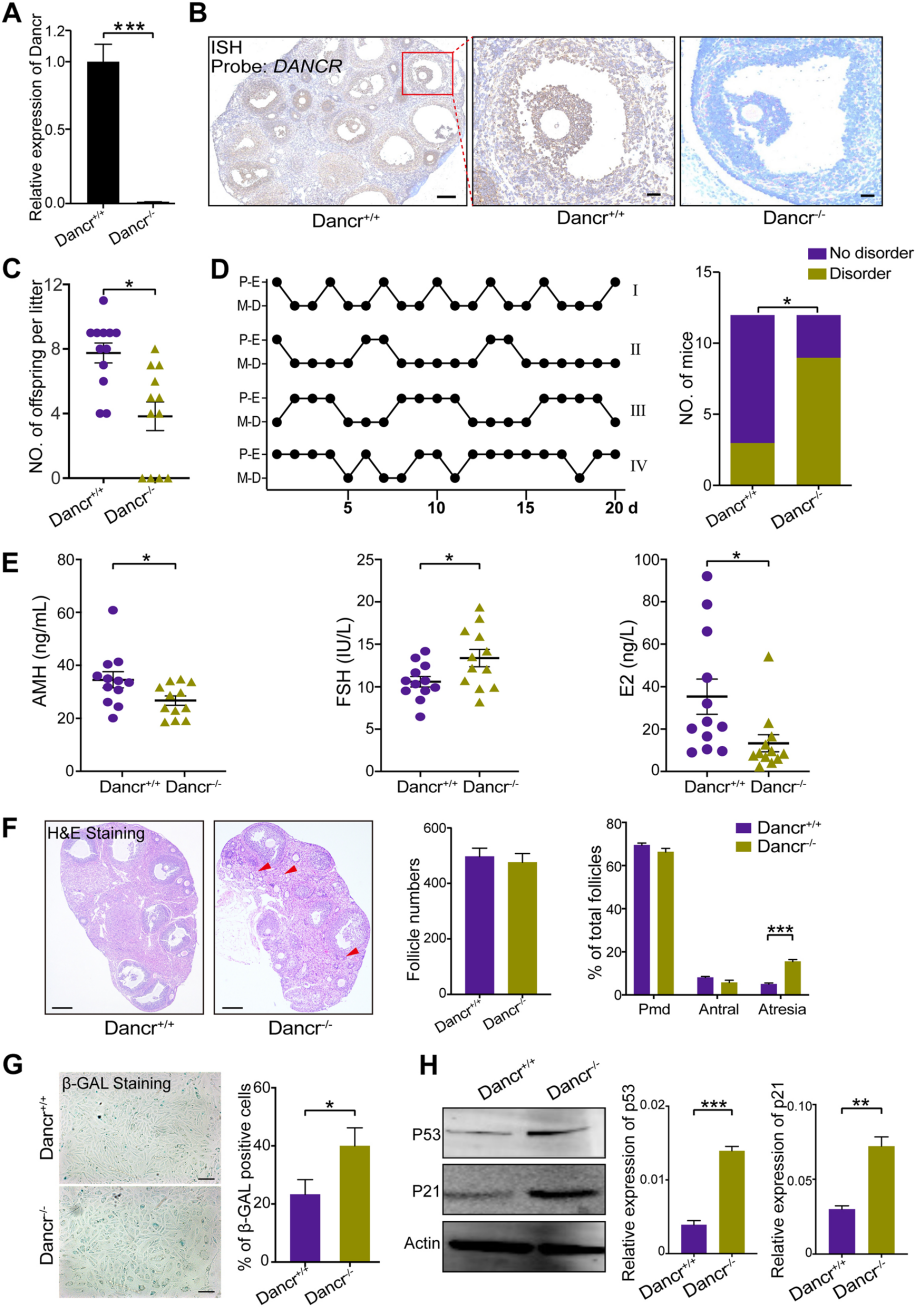
*Dancr*^*−/−*^ mice display impaired fertility and POI phenotypes. **A,** The *Dancr* knockout mice was identified by qPCR. **B,** The ISH results illustrated *Dancr* was specifically expressed in normal ovarian GCs and knocked out in GCs of *Dancr*^*−/−*^ mice. Scale bars: 200 µm (overview), 50 µm (zoom-in). **C,** The live birth number of *Dancr*^+*/*+^ and *Dancr*^*−/−*^ mice per litter. **D,** Left, Four patterns of estrous cycles observed in the study: I, normal cycles; II, disordered cycles with a prolonged diestrus and normal estrus; III, disordered cycles with prolonged estrus and diestrus; IV, disordered cycles with a prolonged estrus and normal diestrus. The *x* axis represents the number of observing days and y axis represents the cycle day in proestrus or estrus (P-E) and metestrus or diestrus (M-D). Right, The ratio of the mice with normal or disordered estrous cycle. **E,** Serum AMH, FSH and E_2_ levels in 8-weeks *Dancr*^+*/*+^ and *Dancr*^*−/−*^ mice. **F,** Left, HE staining sections of mouse ovaries, arrow indicates atresia follicle. Right, Total number of mouse follicles in all serial sections and the proportion of follicles at different stages. Scale bar: 200 µm. Pmd represents primordial follicle; Antral represents antral follicle; Atresia represents atretic follicle. **G,** SA-β-Gal staining of mice primary ovarian granulosa cells and quantify analysis in *Dancr*^*−/−*^ and *Dancr*.^+*/*+^ mice. Scale bar: 50 μm. **H,** Protein levels of p53 and p21 were detected using Western blot analysis. *p < 0.05, ** *p* < 0.01, *** *p* < 0.001

To investigate whether aberrant *Dancr* expression could lead to POI phenotypes and contribute to granulosa cell aging in vivo, *Dancr* conventional knockout mice were constructed by cre-loxp system. qPCR analysis identified the *Dancr*^*−/−*^ mice (Fig. 4A). Further, in situ hybridization (ISH) assays was performed in mice ovarian tissues with DANCR-specific probe. The results demonstrated that DANCR was expressed specifically in ovarian granulosa cells of *Dancr*^+*/*+^ mice but not expressed in *Dancr*^*−/−*^ mice (Fig. 4B). *Dancr*^*−/−*^ mice produced significantly fewer offspring than *Dancr*^+*/*+^ mice per litter, and some even lost their fertility (Fig. 4C). Considering the fertility decline of *Dancr*^*−/−*^ mice, we further assessed the ovarian function of *Dancr*^*−/−*^ mice by multiple methods. About 75% *Dancr*^*−/−*^ mice showed disturbance of estrous cycle, characterized by prolonged estrus, prolonged diestrus or prolonged estrus and diestrus, and the ratio of disordered estrous cycle was significantly higher than *Dancr*^+*/*+^ mice (Fig. 4D). Hormones reflecting ovarian reserve in *Dancr*^*−/−*^ were disturbed compared with *Dancr*^+*/*+^. Concretely, follicle stimulating hormone (FSH) was significantly higher while AMH and basic estradiol (E_2_) was significantly lower in *Dancr*^*−/−*^ group (Fig. 4E). We performed HE-staining with sections of mice ovary tissues and counted the follicle number. It was discovered that the percentage of atresia follicles was sharply increasing in *Dancr*^*−/−*^ mice, but the percentage of primordial follicle and antral follicle was slightly declining (Fig. 4F), which is coincident with the pathological features of POI [4]. Further, we isolated and collected ovarian granulosa cells from sexually mature *Dancr*^*−/−*^ and *Dancr*^+*/*+^ mice separately. SA-β-Gal staining and quantitative analysis revealed that aging GCs appeared more notably in *Dancr*^*−/−*^ mice than the controls (Fig. 4G). Moreover, the protein level of p53 and p21 were also elevated in granulosa cells of *Dancr*^*−/−*^mice (Fig. 4H). In general, *Dancr* knockout in mice is more likely to induce the occurrence of POI and granulosa cells aging.

### DANCR inhibits granulosa cell aging via regulating the interaction of hNRNPC and p53

To elucidate the mechanisms involved in DANCR deficiency promoting granulosa cells aging and POI, HuProt™ human proteome microarray was applied to detect DANCR binding proteins [31, 32]. Volcano plot showed the binding proteins of *DANCR* sense and anti-sense (Suppl. Figure 1A). GO enrichment analysis of DANCR binding proteins revealed the top biological process items focused on RNA regulation process (Suppl. Figure 1B & C). 29 DANCR-binding proteins were screened by Z-Score ≥ 2.8, of which only 4 proteins (RAB2B, ELAVL4, hNRNPC, RBM41) specifically bind to DANCR sense chain, 9 proteins specifically bind to antisense chain, and 16 were non-specifically interacted (Table. S1). The candidate protein heterogeneous nuclear ribonucleoprotein C (hNRNPC) was found highly expressed in ovarian tissue based on the results of NCBI database query. Intriguingly, the protein–protein interaction (PPI) network constructed by STRING indicated that only hNRNPC interacted with p53 (Suppl. Figure 1D). Moreover, previous studies identified the interactions between hNRNPC and p53 protein, and suggested hNRNP family including hNRNPC participated in the p53 regulatory network by interacting with long noncoding RNAs [33, 34]. Thus, we postulated that DANCR interacts with hNRNPC to regulate p53 expression.

**Fig. 5:**
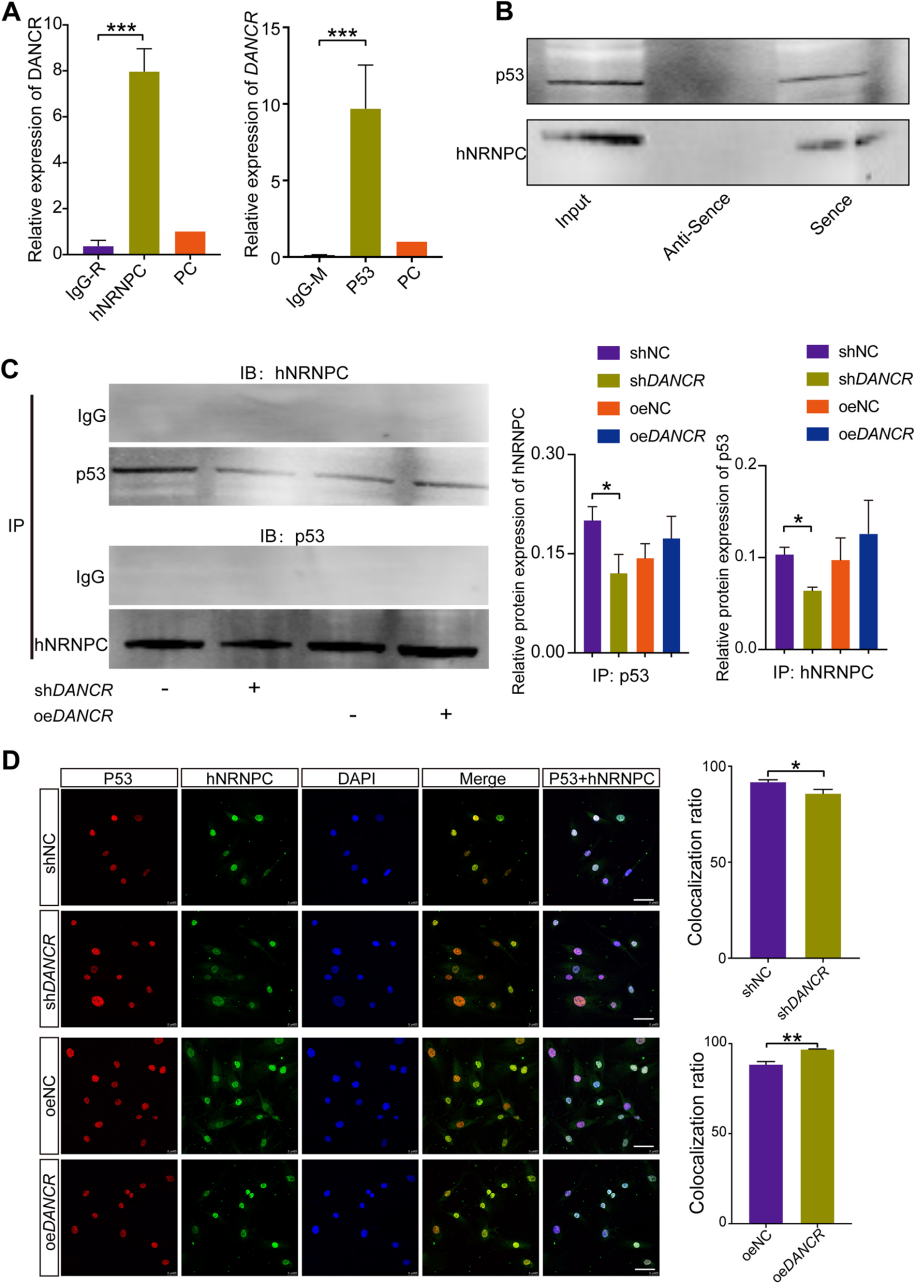
DANCR binds to hNRNPC and p53, and regulates the interaction between hNRNPC and p53. RIP (**A**) and RNA pull down (**B**) assays was performed to detect the DANCR binding to hNRNPC and p53. **C,** The Co-IP results suggested that DANCR knockdown upregulated the p53 protein level and attenuated the interaction between hNRNPC and p53. The relative expression in histogram was calculated by Co-IP samples grayscale value / Input samples grayscale value. **D,** Co-localization fluorescence of hNRNPC and p53 indicated the effects of DANCR on the two proteins binding. Scale bar: 50 μm. *p < 0.05, ** *p* < 0.01, *** *p* < 0.001

The binding of DANCR-hNRNPC and DANCR-P53 were validated in KGN cell lines by RNA pull-down assay and RNA-binding protein immunoprecipitation (Fig. 5A and B). The expression of P53 was upregulated apparently in KGN cells with DANCR knocked down, while the interaction between hNRNPC and P53 significantly decreased. Conversely, overexpression of DANCR could slightly enhance the binding between hNRNPC and P53 (Fig. 5C). In addition, hNRNPC and p53 proteins colocalization staining indicated that DANCR knockdown attenuate the binding of hNRNPC and P53 in the cell nucleus, and on the contrary DANCR overexpression enhanced the binding (Fig. 5D).

**Fig. 6:**
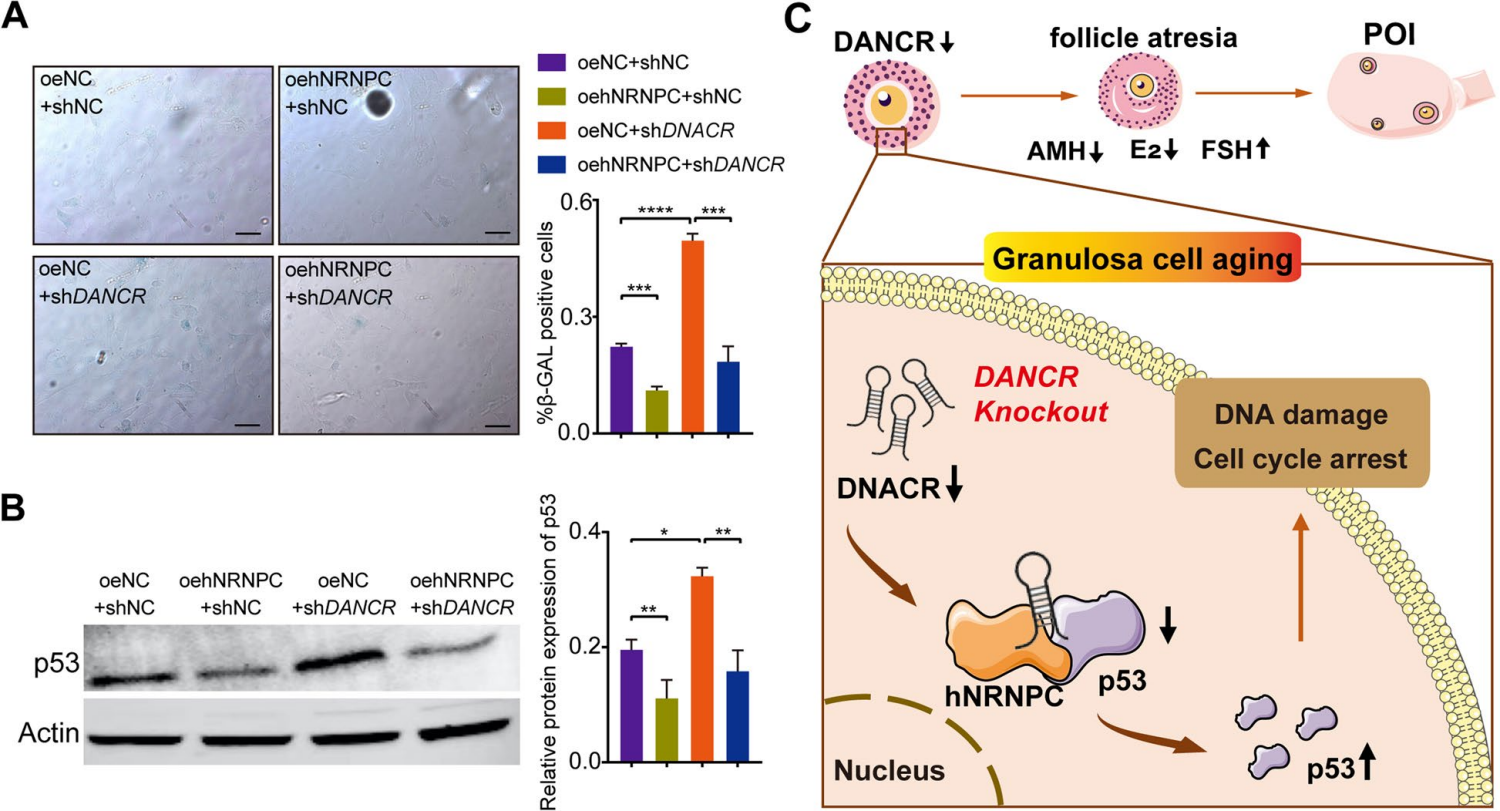
Knockdown of DANCR promotes granulosa cells aging via regulating the interaction of hNRNPC and p53. **A,** SA-β-Gal assays showed the cell aging with different transfection treatments in KGN cells. Knockdown of DANCR (shDANCR group) promoted cell aging, while meanwhile overexpressed hNRNPC (OEhNRNPC + shDANCR group) could relieve cell aging. Scale bar: 50 μm. **B,** Protein level of p53 with different transfection treatments in KGN cells. The increased p53 expression when DANCR knockdown, was significantly reduced with overexpressed hNRNPC. shNC and OENC serve as negative control. *p < 0.05, ** *p* < 0.01, *** *p* < 0.001, **** *p* < 0.0001. **C,** A schematic graph illustrating the role of DANCR in POI. Knockdown of DANCR upregulates the p53 protein level through alleviating the binding of p53 and hNRNPC, which enhances ovarian granulosa cells aging, follicles atresia and hormone disorders, eventually casing POI development

To identify whether downregulating DANCR promoted the granulosa cells aging by regulating the interaction of hNRNPC and P53, shDANCR and overexpressed hNRNPC (OEhNRNPC) were transfected into KGN cells. As the results show, when transfected with shDANCR alone, it upregulated the p53 expression and induced granulosa cells aging (Fig. 6A and B). Subsequently transfected with shDANCR + OEhNRNPC, the expression of p53 was decreased and the cell aging was notably alleviated compared with shDANCR group (Fig. 6A and B). In summary, DANCR knockdown reduces the binding of hNRNPC and P53 and increases the protein level of p53, which accelerates granulosa cell aging eventually.

In this study, we explored the function and mechanism of the lncRNA-DANCR in the pathogenesis of POI. The binding of hNRNPC and p53 was inhibited when DANCR knockdown, which contributed to the accumulation of p53 and granulosa cells aging. Conclusively, DANCR knockdown may induce accelerated senescence of granulosa cells, aggravate follicular atresia and hormone disorders and eventually cause POI (Fig. 6C).

## Discussion

POI is a disorder with premature decline of ovarian physiological function which impairs women fertility severely, but more than half cases are idiopathic and the etiology is still unknown. Most studies focused on exploring the protein-coding genes involved in POI while few research on the roles of non-coding RNA, especially lncRNAs. Wand et al. [16]. confirmed the first lncRNA involved in POI, that is HCP5 transcriptionally regulating MSH5 and DNA damage repair via YB1 in GCs. Recently, several lncRNAs were discovered to exert their functions by regulating GCs proliferation or apoptosis in POI [18, 20, 21]. In this study, we firstly report that decreased expression of lncRNA DANCR participate in GCs aging and POI process.

DANCR has been widely regarded as a valuable oncogenic lncRNA whose dysregulated expression was found in a variety of malignancies, including hepatocellular carcinoma and ovarian cancer and so on [35–37]. However, as a relatively high-expressed lncRNA in ovary tissue, its roles in ovarian function regulation remains to be unclear. Here, we confirmed that, DANCR was expressed in human and mice ovarian GCs and coincident with the result of RNA-seq [26]. And our study claimed that the expression of DANCR in granulosa cells of POI patients was significantly downregulated. Moreover, DANCR knockout mice present POI related phenotypes including fertility declining, disturbed estrous cycle and abnormal endocrine hormone. The histological analysis suggests knockout of DANCR promotes follicular atresia, a crucial part of POI pathogenesis. Previous studies have proved that GCs death, such as cell apoptosis, necroptosis and autography, affects the follicular atresia [38–41]. Transcriptome profiling of GCs indicated the overall processes influenced by transcription factor p53 were predicted to be activated in atretic follicles [38]. In the study, deficiency of DANCR inhibited GCs proliferation and accelerate GCs aging with phenotypes such as G1 arrest, DNA damage and p53 upregulation in vitro and in vivo, (Figs. 3, 4 and 5). In consequence, DANCR participates in POI through regulating granulosa cells aging.

LncRNAs often exert their functions by binding one or more RNA-binding proteins acting as decoys, scaffolds, guides, and signals to regulate gene expression and cellular physiology [42, 43]. Human proteome microarray detected four proteins that specifically bound to the sense chain of DANCR in our study. Based on the results of bioinformatic analysis, hNRNPC was predicted to be involved in the DANCR regulating granulosa cells aging. And the further investigation clarified that, knockdown of DANCR attenuated the combination of hNRNPC with p53, resulting in increased p53 protein level and GCs aging. Similarly, a subject has found that lncRNA SNHG1 competes with p53 for hNRNPC binding, which upregulates p53 expression and promotes p53-dependent apoptosis [33]. hNRNPC, as an RNA binding protein belonging to the hNRNPs subfamily ubiquitously expressed, was involved in the initial steps of spliceosome assembly and pre-mRNA splicing [44]. hNRNPC1/C2 proteins interacting with a cis-regulatory element within p53 mRNA regulates the expression p53 and contributes to the apoptotic process [45]. Through RIP and RNA pull-down assays, we find that DANCR respectively binds to hNRNPC and p53, therefore indicate that DANCR may serve as a bridge to connect hNRNPC and p53. There’s limitation of the study yet that the detail mechanisms of DANCR regulating hNRNPC-p53 protein interactions remains to be elucidated in future research.

## Conclusions

Overall, we claim that lncRNA DANCR is involved in negatively regulating the granulosa cells aging and POI development. The expression of DANCR decreases in POI patients’ granulosa cells and *Dancr*^*−/−*^ mice present POI related phenotypes. Mechanistic investigation finds that, DANCR knockdown attenuates the interaction between hNRNPC and p53, which enhances p53 protein level and induces granulosa cells aging. The senescence of granulosa cells aggravates follicle atresia and leads to POI. This study provides the potential of DANCR as a POI biomarker, and it may become a new therapeutic target for POI.

## Materials and methods

### Patient enrollment and GC samples collection

This study was approved by the Ethics Committee of Changzheng Hospital Affiliated with Naval Medical University (No. 2018SLYS1). All patients enrolled were undergoing in vitro fertilization-embryo transfer (IVF) or intracytoplasmic sperm injection (ICSI) treatments in reproductive medicine center and signed informed consent. Our study included 8 patients diagnosed as POI according to the ESHRE guideline [46], and 10 infertile women because of tubal or male factor, with normal ovarian response, regular menses, normal basal hormone levels and BMI 19–24, as the control group. Because POI patients had been treated with hormone replacement therapy, the basic hormone level and menstrual cycle have recovered, and the low AMH level (AMH < 1.5 ng/ml) reflecting poor ovarian reserve was the key inclusion criteria for POI. The major anthropometric variables and endocrine parameters of the women are listed in Table 1. All the human ovarian GC samples were collected from the discarded products of transvaginal oocyte retrieval and isolation. The preparation of GC samples in the laboratory followed our previous procedure [47].

### Animals

*Dancr*^*fl/fl*^ and *CMV-Cre* mice were constructed and purchased from Biocytogen Pharmaceuticals (Beijing) Co.,Ltd. *Dancr* gene is on the positive strand of chromosome 5, with a full length of 1.25 kb (NCBI ID: 70,036). The sgRNAs are designed to be approximately 2 kb upstream of *Dancr* and 500 kb downstream of *Dancr* in a non-conserved region. *Dancr*^*fl/fl*^ mice was screened with genotype verification. *CMV-Cre* mice express Cre recombinase under the control of a human cytomegalovirus (CMV) promoter, active in most cells and tissues. *Dancr*^*fl/fl*^ mice mated with *CMV-Cre* mice. *Dancr* gene knockout mice (*Dancr*^*−/−*^) were obtained after three generations and *Dancr*^+*/*+^ mice were a negative control for follow-up experiments. In other experiments, C57BL/6 mice, aged 8 weeks and 36 weeks, were purchased from SLAC Laboratory Animal Company. The following primers used for *Dancr*^*−/−*^ genotyping were 5′loxP-F: GACCTCTAGCAAGTAGACCAGGGGA; 5′loxP-R: GTCTGTTATCTGGATCCTGTAGTCCTGG;3′loxP-F:GACCTCTAGCAAGTAGACCAGGGGA and 3′loxP-R:TCGGGCTAGTGCAGGTGTTAGCAGGT [48]. Animal were maintained at a temperature of 25 °C and a humidity of 50% to 60%, with a 12:12 h light–dark cycle with ad libitum water and food. All experiments were approved by the Ethics Committee of Changzheng Hospital Affiliated with Naval Medical University.

### Estrous cycle and cage mating

8-week-old *Dancr*^*−/−*^ or *Dancr*^+*/*+^ female mice were performed with vaginal smear once a day lasting for 20 days, and each group contained 12 mice. The slides were fixed with 90% ethanol for 5 min, Giemsa stained for 15 min, washed gently and dried. The types of cells in vaginal smears were evaluated by optical microscopy. With cytological evaluation, proportions of keratinized epithelial cell, nucleated epithelial cell and leukocytes as a criterion to determine the phase of estrous cycle [49]. There are three types of disordered estrous cycle in our study: a) prolonged estrous with normal diestrus; b) prolonged diestrus with normal estrous; c) prolonged estrous and diestrus.

Each mouse was weighed first, and there was no significant difference in mean weight between the *Dancr*^*−/−*^ and *Dancr*^+*/*+^ groups. Each group contains 12 mice. 8-week-old female *Dancr*^*−/−*^ mice and *Dancr*^+*/*+^ mice were 1:1 mated with 8-week-old male mice in the cage randomly. The next morning (day post coitum [DPC] 0.5), vaginal plug was detected. The female mice with vaginal plug were thought to be pregnant. If there’s not vaginal plug, the male mice were changed cages for further mating. The pregnant female mice were fed in the separate cages.

### Mouse primary ovarian granulosa cells isolation

Both ovaries of sacrificed mice were carefully removed and cleaned with PBS gently. Place the ovaries into a dish under a stereo microscope for further operation. The antral follicles were punctured with a 1 ml disposable syringe and granulosa cells were extruded slowly. All of the granulosa cells were removed into EP tubes using a 10 µl pipette and digested with hyaluronidase at room temperature for 10 min. After washing with PBS for 3 times, the primary granulosa cells were prepared for subsequent experiments.

### Cell lines and cell culture

The human ovarian granulosa cell lines KGN and COV434 kept in our laboratory with STR (short tandem repeat) identification were cultured in DMEM (HyClone, USA) supplemented with 1% fetal bovine serum (Gibco, USA) and 100 IU/mL penicillin–streptomycin at 37 °C in a humidified atmosphere of 5% CO_2_ and 95% air.

### Enzyme linked immunosorbent assay (ELISA)

Mice whole blood was collected from the eye socket veins, and then balanced at 4 °C for 1 h, followed by centrifugation at 3000 rpm for 20 min. The upper serum was transferred and storage at -20 °C/-80 °C. The mice serum AMH, estradiol (E2), and FSH were measured with ELISA kits (Demeditec, Germany, DE1288/DE2693 & JianglaiBio, China, JL20476) according to manufacturer’s instructions.

### Hematoxylin and eosin (HE) staining

The unilateral ovary tissues collected from 8-week-old *Dancr*^*−/−*^ and *Dancr*^+*/*+^ mice were fixed in 4% paraformaldehyde (Sigma), dehydrated, and paraffin-embedded. Then the tissues were cut into continuous 4-μm-thick sections. One slide selected at interval of five, totally 20 sections per ovary tissue sample were obtained for HE staining. The follicles at each stage were counted separately and total follicles were counted.

### Fluorescence in situ hybridization (ISH/FISH)

ISH/FISH probes for DANCR (Human:5’-CY3-TGGCTTGTGCCTGTAGTTGTCAACCTGCGC-CY3-3’; Mouse:5’-DIG-AGTGGGACATGAAGAAGGGGTGGGGCA-DIG-3’) were synthesized by Wuhan Servicebio Company. ISH/FISH was performed according to the protocol of FISH assay kit. Representative images were captured under a fluorescence microscope.

### RNA extraction and quantitative real-time PCR (qPCR)

Total RNA from cells or tissues were extracted with TRIzol reagent and RNeasy Mini Kit (Qiagen), and were subsequently reverse transcribed into cDNA by PrimerScript™ RT Master Mix Kit (Takara). RNA quantitation was performed with TB Green Premix EX Taq kit (Takara) on the ABI QuantStudio6 system according to manufacturer’s protocol. The primers used for amplification of DANCR are listed in Supplementary Table 2.

### Lentivirus vector and gene transduction

The lentiviral vector system pLKD-CMV-G&PR-U6 was performed to gene knockdown and overexpression in granulosa cell lines (KGN or COV434). The DANCR shRNA/overexpressed lentiviral vectors were constructed by OBiO Technology Corporation. (DANCR shRNA, 5′-GCAGCTGCCTCAGTTCTTA-3′; NC shRNA, 5′-TTCTCCGAACGTGTCACGT-3′). Cell infection was conducted as follows: the cultures were transfected with lentiviral particles according to OBiO Kit. After infection, the stable transfectants were selected using 2 mg/mL puromycin in the presence of 10% FBS for 72 h. The efficiency of DANCR downregulation in KGN and COV434 cells was confirmed by real-time qPCR. The qPCR primers were listed in the Table S2.

### Cell proliferation and aging assays

Cell viability and proliferation was detected by cell counting kit (CCK)-8 (Dojindo, Japan) and 5-Ethynyl-20-Deoxyuridine (EdU) assay (RiboBio, China). Cell cycle was detected by flow cytometry with cells incubated with DNA staining solution (PBS containing 20 μg/ml RNase A and 20 μg/ ml PI) at 37 °C for 30 min in dark place. For cell aging evaluation, senescent-associated β-gal (SA-β-Gal) and γ-H2AX staining were performed, with imagines captured under microscopy.

### Proteome microarray

The sense and anti-sense lncRNA DANCR for protein microarray screening were synthesized labeling with Cy5 dye. The HuProt microarray from Wayen biotechnologies, comprised of more than ~ 20,000 full length human proteins with N-terminal GST-fusions, were performed according to the manufacturer’s instructions. Specifically, proteome microarrays were blocked for 1 h at room temperature. The lncRNA was placed in blocking buffer and incubated with the proteome microarray at room temperature for 1 h. The microarrays were washed three times for 5 min each time with TBST and then three times with Milli-Q water. Dried slides were scanned using GenePix 4000B, and images were analyzed using GenePix Pro 6.0. Results are presented in Supplementary File (Table S1).

### Western blotting (WB) and Co-immunoprecipitation (Co-IP)

Cells were lysed with radioimmunoprecipitation assay (RIPA) buffer (Beyotime, Shanghai, China) containing Protease/Phosphatase Inhibitor Cocktail (Epizyme, Shanghai, China) in ice-cold. The abundance of total protein was determined using a BCA Protein Assay Kit (Beyotime, Shanghai, China). Equal amount protein per lane was loaded onto an SDS–polyacrylamide gel, electrophoresed and transferred to polyvinylidene fluoride (PVDF) membranes (Millipore, USA). After blocking with 5% nonfat milk for 1 h, the membranes were incubated with diluted primary antibodies γ-H2AX (Millipore, USA, 05–636-AF488); p53 (CST, USA, 2524); hNRNPC (Novusbio, USA, SN0652); P21(Affinity, USA, AF6290); β-actin (CST, USA, 4967/3700), GAPDH (CST, USA, 5174) overnight at 4 °C, then washed and incubated with indicated secondary antibodies (IRDye 680RD, IRDye800CW (Licor, USA, #c50408-02 #c50331-05). The Odyssey infrared scanner (Li-COR Biosciences, Nebraska, USA) was used to detect the protein bands and β-actin served as a loading control.

For Co-IP, the total protein was extracted as the WB protocol above. Antibodies for IP was incubated with magnetic beads (Thermo, USA, 88,802) for 40 min and washed with PBS for three times. Extracted protein and magnetic beads were incubated overnight at 4 °C. The magnetic beads were washed with PBS for 5 times, and 80 μl PBS was added to re-suspend the beads. After protein denaturation at 100 °C for 10 min, magnetic beads were removed by magnetic rack, and the protein sample was completely prepared for further WB detection.

### RNA immunoprecipitation (RIP)

RNA-binding protein immunoprecipitation assays were performed by a Magna RIP Kit (Millipore, USA) according to the manufacturer’s methods. Cells were lysed in RIP lysis buffer, and magnetic beads with HNRNPC (Novusbio, #SN0652), P53 (CST, #2524) or IgG antibody were prepared. The immunoprecipitation reactions were carried out by incubating the RIP lysate and the beads-antibody complex together with rotation overnight at 4 °C. Immunoprecipitated RNAs were purified and analyzed by qPCR and normalized to the input control.

### RNA pull down

DANCR plasmid was synthesized by OBiO Technology Corporation and extracted with plasmid extraction kit (Tiangen, China). SP6 and T7 enzymes were added overnight at 37 °C for enzyme digestion and transcription in vitro was performed with the kit. Biotin was labeled on the DANCR-RNA transcribed and incubated with Dynabeads (Invitrogen, USA, 65,001). Cell protein samples were prepared in advance and incubated with the Dynabeads-RNA complex at 4 °C overnight. The enriched protein was eluted and preserved for Western Blot to verify the protein lane.

### Statistical analysis

Statistical analyses were performed using GraphPad Prism 8.0 and image quantitative analyses were performed with Image J. Data are presented as the mean ± SEM. Student’s t-test for two groups and one-way ANOVA analysis for three or more groups were used for group comparisons. Fisher's precision probability test was used to analyze the proportion of disordered estrous cycles. Nonparametric t-test was used for the analysis of mice pregnancy rate. All the experiments were repeated more than three times, and *p* < 0.05 was considered statistically significant.

## Supplementary Information

**Additional file 1: Supplementary Figure S1. **Bioinformatics analysis of protein microarray data.

**Additional file 2:** **Supplementary Table 1.** RNA binding proteins of *DANCR *via human proteomics chip. **Supplementary Table 2.** The primers for qPCR.

## Data Availability

The datasets used and analysed during the current study are available from the corresponding author on reasonable request.

## Abbreviations

POI: Premature ovarian insufficiency
GC: Granulosa cell
lncRNA: Long non-coding RNA
DANCR: Differentiation antagonizing non-protein coding RNA
hNRNPC: Heterogeneous nuclear ribonucleoprotein C
IVF: In vitro fertilization
ICSI: Intracytoplasmic sperm injection
RIP: RNA immunoprecipitation
ISH: In situ hybridization
FISH: Fluorescence in situ hybridization
SASP: Senescence-associated secretory phenotype

## References

[1] Coulam CB, Adamson SC, Annegers JF (1986). Incidence of premature ovarian failure. Obstet Gynecol.

[2] Golezar S, Ramezani Tehrani F, Khazaei S, Ebadi A, Keshavarz Z (2019). The global prevalence of primary ovarian insufficiency and early menopause: a meta-analysis. Climacteric.

[3] Jiao X, Zhang H, Ke H, Zhang J, Cheng L, Liu Y (2017). Premature Ovarian Insufficiency: Phenotypic Characterization Within Different Etiologies. J Clin Endocrinol Metab.

[4] De Vos M, Devroey P, Fauser BC (2010). Primary ovarian insufficiency. Lancet.

[5] Veitia RA (2020). Primary ovarian insufficiency, meiosis and DNA repair. Biomed J.

[6] Yu YS, Sui HS, Han ZB, Li W, Luo MJ, Tan JH (2004). Apoptosis in granulosa cells during follicular atresia: relationship with steroids and insulin-like growth factors. Cell Res.

[7] Yeung CK, Wang G, Yao Y, Liang J, Tenny Chung CY, Chuai M (2017). BRE modulates granulosa cell death to affect ovarian follicle development and atresia in the mouse. Cell Death Dis.

[8] Wang J, Chu K, Wang Y, Li J, Fu J, Zeng YA (2021). Procr-expressing granulosa cells are highly proliferative and are important for follicle development. iScience.

[9] E F, Zhang H, Yin W, Wang C, Liu Y, Li Y (2021). CPEB3 deficiency in mice affect ovarian follicle development and causes premature ovarian insufficiency. Cell Death Dis.

[10] Guo L, Liu X, Chen H, Wang W, Gu C, Li B (2022). Decrease in ovarian reserve through the inhibition of SIRT1-mediated oxidative phosphorylation. Aging (Albany NY).

[11] Jiang Z-x, Wang Y-n, Li Z-y, Dai Z-h, He Y, Chu K (2021). The m6A mRNA demethylase FTO in granulosa cells retards FOS-dependent ovarian aging. Cell Death Dis.

[12] Tilly JL, Tilly KI, Perez GI (1997). The genes of cell death and cellular susceptibility to apoptosis in the ovary: a hypothesis. Cell Death Differ.

[13] Wang XY, Qin YY (2019). Long non-coding RNAs in biology and female reproductive disorders. Front Biosci (Landmark Ed).

[14] Pankiewicz K, Laudanski P, Issat T (2021). The Role of Noncoding RNA in the Pathophysiology and Treatment of Premature Ovarian Insufficiency. Int J Mol Sci.

[15] Yao G, He J, Kong Y, Zhai J, Xu Y, Yang G (2019). Transcriptional profiling of long noncoding RNAs and their target transcripts in ovarian cortical tissues from women with normal menstrual cycles and primary ovarian insufficiency. Mol Reprod Dev.

[16] Wang X, Zhang X, Dang Y, Li D, Lu G, Chan WY (2020). Long noncoding RNA HCP5 participates in premature ovarian insufficiency by transcriptionally regulating MSH5 and DNA damage repair via YB1. Nucleic Acids Res.

[17] Li D, Wang X, Dang Y, Zhang X, Zhao S, Lu G (2021). lncRNA GCAT1 is involved in premature ovarian insufficiency by regulating p27 translation in GCs via competitive binding to PTBP1. Mol Ther Nucleic Acids.

[18] Li D, Wang X, Li G, Dang Y, Zhao S, Qin Y (2021). LncRNA ZNF674-AS1 regulates granulosa cell glycolysis and proliferation by interacting with ALDOA. Cell Death Discov.

[19] Li D, Xu W, Wang X, Dang Y, Xu L, Lu G (2021). lncRNA DDGC participates in premature ovarian insufficiency through regulating RAD51 and WT1. Mol Ther Nucleic Acids.

[20] Zheng C, Liu S, Qin Z, Zhang X, Song Y (2021). LncRNA DLEU1 is overexpressed in premature ovarian failure and sponges miR-146b-5p to increase granulosa cell apoptosis. J Ovarian Res.

[21] Zhang L, Mao B, Zhao X, Yuan Y, Wang W, Lin S (2022). Translation regulatory long non-coding RNA 1 (TRERNA1) sponges microRNA-23a to suppress granulosa cell apoptosis in premature ovarian failure. Bioengineered.

[22] Kretz M, Webster DE, Flockhart RJ, Lee CS, Zehnder A, Lopez-Pajares V (2012). Suppression of progenitor differentiation requires the long noncoding RNA ANCR. Genes Dev.

[23] Zhang Z, Shu L, Hu M, Zhou X, Yang F, Zhou XH (2021). Emerging role of lncRNA DANCR in progenitor cells: beyond cancer. Eur Rev Med Pharmacol Sci.

[24] Jin SJ, Jin MZ, Xia BR, Jin WL (2019). Long Non-coding RNA DANCR as an Emerging Therapeutic Target in Human Cancers. Front Oncol.

[25] Wang M, Gu J, Zhang X, Yang J, Zhang X, Fang X (2021). Long Non-coding RNA DANCR in Cancer: Roles, Mechanisms, and Implications. Front Cell Dev Biol.

[26] Fagerberg L, Hallstrom BM, Oksvold P, Kampf C, Djureinovic D, Odeberg J (2014). Analysis of the human tissue-specific expression by genome-wide integration of transcriptomics and antibody-based proteomics. Mol Cell Proteomics.

[27] Xu X, Chen X, Zhang X, Liu Y, Wang Z, Wang P (2017). Impaired telomere length and telomerase activity in peripheral blood leukocytes and granulosa cells in patients with biochemical primary ovarian insufficiency. Hum Reprod.

[28] Sheekey E, Narita M. p53 in senescence - it's a marathon not a sprint. FEBS J. 2021. 10.1111/febs.16325. 10.1111/febs.16325 34921507

[29] Rufini A, Tucci P, Celardo I, Melino G (2013). Senescence and aging: the critical roles of p53. Oncogene.

[30] Campisi J (2005). Senescent cells, tumor suppression, and organismal aging: good citizens, bad neighbors. Cell.

[31] Siprashvili Z, Webster DE, Kretz M, Johnston D, Rinn JL, Chang HY (2012). Identification of proteins binding coding and non-coding human RNAs using protein microarrays. BMC Genomics.

[32] Barry G, Briggs JA, Vanichkina DP, Poth EM, Beveridge NJ, Ratnu VS (2014). The long non-coding RNA Gomafu is acutely regulated in response to neuronal activation and involved in schizophrenia-associated alternative splicing. Mol Psychiatry.

[33] Shen Y, Liu S, Fan J, Jin Y, Tian B, Zheng X (2017). Nuclear retention of the lncRNA SNHG1 by doxorubicin attenuates hnRNPC-p53 protein interactions. EMBO Rep.

[34] Zarnack K, König J, Tajnik M, Martincorena I, Eustermann S, Stévant I (2013). Direct competition between hnRNP C and U2AF65 protects the transcriptome from the exonization of Alu elements. Cell.

[35] Yuan SX, Wang J, Yang F, Tao QF, Zhang J, Wang LL (2016). Long noncoding RNA DANCR increases stemness features of hepatocellular carcinoma by derepression of CTNNB1. Hepatology.

[36] Lin X, Yang F, Qi X, Li Q, Wang D, Yi T (2019). LncRNA DANCR promotes tumor growth and angiogenesis in ovarian cancer through direct targeting of miR-145. Mol Carcinog.

[37] Thin KZ, Liu X, Feng X, Raveendran S, Tu JC (2018). LncRNA-DANCR: A valuable cancer related long non-coding RNA for human cancers. Pathol Res Pract.

[38] Hatzirodos N, Hummitzsch K, Irving-Rodgers HF, Harland ML, Morris SE, Rodgers RJ (2014). Transcriptome profiling of granulosa cells from bovine ovarian follicles during atresia. BMC Genomics.

[39] Regan SLP, Knight PG, Yovich JL, Leung Y, Arfuso F, Dharmarajan A (2018). Granulosa Cell Apoptosis in the Ovarian Follicle-A Changing View. Front Endocrinol (Lausanne).

[40] Zhou J, Peng X, Mei S (2019). Autophagy in Ovarian Follicular Development and Atresia. Int J Biol Sci.

[41] Chaudhary GR, Yadav PK, Yadav AK, Tiwari M, Gupta A, Sharma A (2019). Necroptosis in stressed ovary. J Biomed Sci.

[42] Ferrè F, Colantoni A, Helmer-Citterich M (2016). Revealing protein-lncRNA interaction. Brief Bioinform.

[43] Kopp F, Mendell JT (2018). Functional Classification and Experimental Dissection of Long Noncoding RNAs. Cell.

[44] Choi YD, Grabowski PJ, Sharp PA, Dreyfuss G (1986). Heterogeneous nuclear ribonucleoproteins: role in RNA splicing. Science.

[45] Christian KJ, Lang MA, Raffalli-Mathieu F (2008). Interaction of heterogeneous nuclear ribonucleoprotein C1/C2 with a novel cis-regulatory element within p53 mRNA as a response to cytostatic drug treatment. Mol Pharmacol.

[46] Webber L, Davies M, Anderson R, Bartlett J, Braat D, Cartwright B (2016). ESHRE Guideline: management of women with premature ovarian insufficiency. Hum Reprod.

[47] Dong JP, Dai ZH, Jiang ZX, He Y, Wang L, Liao QY (2019). CD24: a marker of granulosa cell subpopulation and a mediator of ovulation. Cell Death Dis.

[48] Gan X, Ding D, Wang M, Yang Y, Sun D, Li W (2022). DANCR deletion retards the initiation and progression of hepatocellular carcinoma based on gene knockout and patient-derived xenograft in situ hepatoma mice model. Cancer Lett.

[49] Cora MC, Kooistra L, Travlos G (2015). Vaginal Cytology of the Laboratory Rat and Mouse: Review and Criteria for the Staging of the Estrous Cycle Using Stained Vaginal Smears. Toxicol Pathol.

