# Supplementary figures and images for: LncRNA DANCR counteracts premature ovarian insufficiency by regulating the senescence process of granulosa cells through stabilizing the interaction between p53 and hNRNPC
Source: J Ovarian Res. 2023 Feb 18;16:41. doi: 10.1186/s13048-023-01115-3 (PMC9938559; doi:10.1186/s13048-023-01115-3)

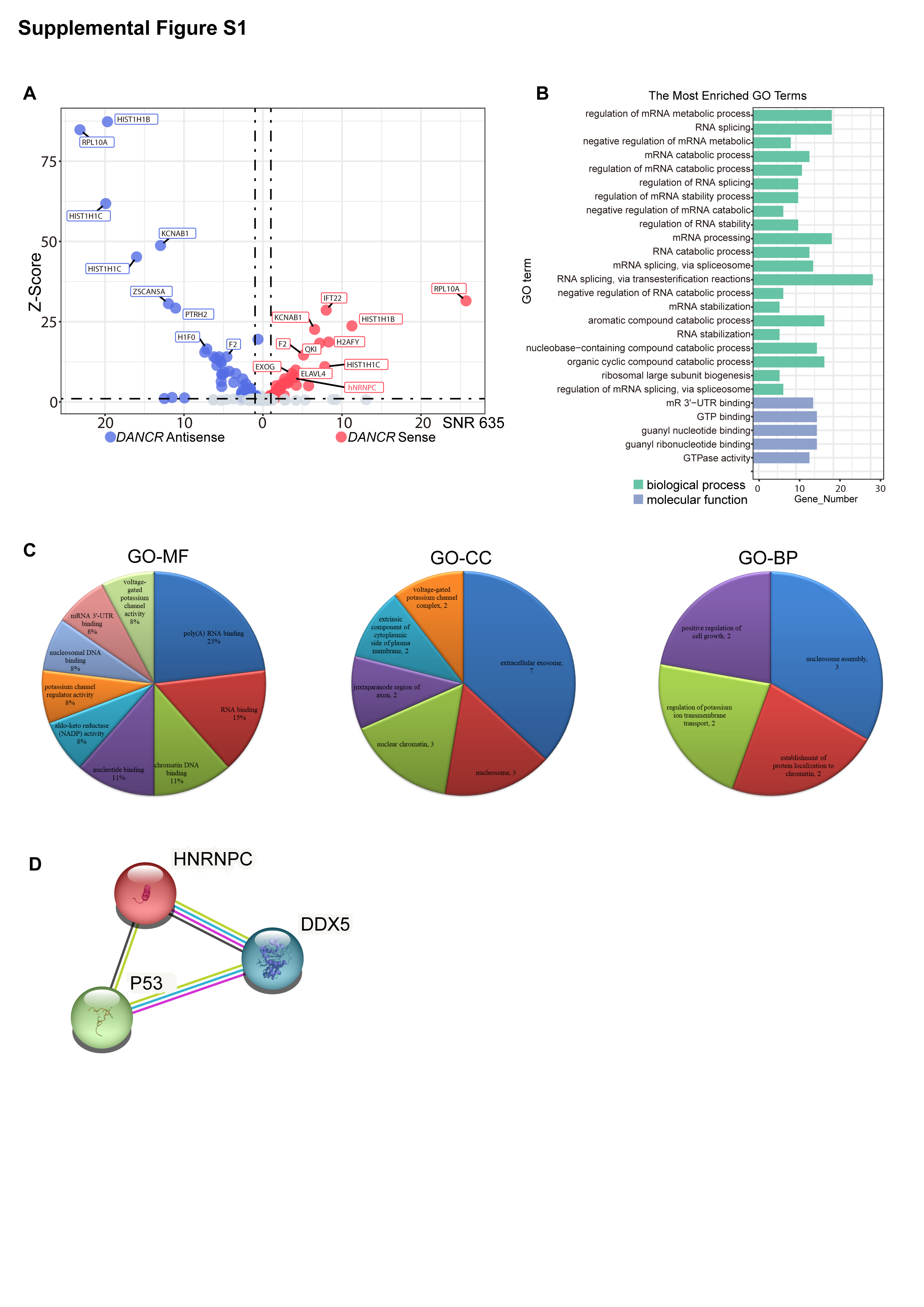

Supplement: Supplementary file 1 — Additional file 1: Supplementary Figure S1. Bioinformatics analysis of protein microarray data. [file 13048_2023_1115_MOESM1_ESM.jpg]
