# Supplementary material for: LncRNA DANCR counteracts premature ovarian insufficiency by regulating the senescence process of granulosa cells through stabilizing the interaction between p53 and hNRNPC
Source: J Ovarian Res. 2023 Feb 18;16:41. doi: 10.1186/s13048-023-01115-3 (PMC9938559; doi:10.1186/s13048-023-01115-3)
Supplement: Supplementary file 2 — Additional file 2: Supplementary Table 1. RNA binding proteins of DANCR via human proteomics chip. Supplementary Table 2. The primers for qPCR. [file 13048_2023_1115_MOESM2_ESM.docx]

**Supplementary Table 1. RNA binding proteins of *DANCR* via human proteomics chip**

| Name | ID |
| --- | --- |
| Sense |  |
| RAB2B | JHU03618.P038B11 |
| ELAVL4 | JHU05591.P059G09 |
| hNRNPC | JHU05226.P055E09 |
| RBM41 | JHU05440.P057C12 |
| anti-Sense |  |
| PGF | JHU02756.P029D09 |
| PTRH2 | JHU02660.P028D06 |
| ZNF385B | JHU05661.P059A10 |
| H1F0 | JHU05317.P056B04 |
| HIST1H1A | JHU10506.P110B10 |
| WISP2 | JHU14066.P147E11 |
| SRSF2 | JHU14049.P147F11 |
| EZR | JHU16385.P172B05 |
| PA2G4 | JHU02175.P229A10 |
| common elements |  |
| RPL10A | JHU02666.P028F07 |
| ZSCAN5A | JHU02496.P026E03 |
| HIST1H1C | JHU02441.P026E04 |
| IFT22 | JHU00265.P003B01 |
| CHMP5 | JHU04436.P047G11 |
| H2AFY | JHU05403.P057H09 |
| KCNAB2 | JHU07151.P075D01 |
| KCNAB1 | JHU05510.P058B02 |
| QKI | JHU08508.P089F11 |
| EXOG | JHU10873.P114E05 |
| F2 | JHU13729.P144G07 |
| KCNAB1 | JHU13741.P144G08 |
| QKI | JHU15165.P159F04 |
| QKI | JHU11870.P124C06 |
| MEX3B | JHU15450.P162D08 |
| HIST1H1B | JHU18560.P213H11 |

**Supplementary Table 2. The primers for qRT-PCR**

| Primer name | Sequence (5' to 3') |
| --- | --- |
| HU-*DANCR*-F | GCGCCACTATGTAGCGGGTT |
| HU-*DANCR*-R | TCAATGGCTTGTGCCTGTAGTT |
| mus-*DANCR*-F | AAACCCGTGACTGAATGGCT |
| mus-*DANCR*-R | TCACATGGCCCTCACTTCAC |

**Supplementary Figure S1 Legend**

**A,** Volcano plot of the binding proteins of *DANCR* sense and anti-sense. **B,** GO enrichment analysis of *DANCR* binding proteins screened by Z-Score≥0.5. **C,** GO enrichment analysis of *DANCR* sense binding proteins screened by Z-Score≥2.8. **D,** The protein-protein interaction (PPI) network between HNRNPC and P53 constructed by STRING database.
